# Intra-ampullary and Periampullary Carcinoma: Clinicopathological Comparison and Survival Outcomes

**DOI:** 10.7759/cureus.67030

**Published:** 2024-08-16

**Authors:** Atif A Hashmi, Ramla Ali, Syed Sualeh Jamal, Sumbal Zafar, Shamail Zia, Fazail Zia, FNU Anjali, Sanjay Kirshan Kumar, Muhammad Irfan

**Affiliations:** 1 Pathology, Liaquat National Hospital and Medical College, Karachi, PAK; 2 Internal Medicine, Liaquat National Hospital and Medical College, Karachi, PAK; 3 Internal Medicine, Baqai Medical University, Karachi, PAK; 4 Pathology, Jinnah Sindh Medical University, Karachi, PAK; 5 Internal Medicine, Sakhi Baba General Hospital, Sukkur, PAK; 6 Internal Medicine, Bahria University Medical and Dental College, Karachi, PAK; 7 Statistics, Liaquat National Hospital and Medical College, Karachi, PAK

**Keywords:** pancreatic carcinoma, intestinal, pancreatobiliary, periampullary carcinoma, intra-ampullary carcinoma, ampullary carcinoma

## Abstract

Introduction

The ampulla of Vater is a structure in the duodenal wall in which the biliary and pancreatic ducts open. Malignant epithelial tumors arising at this site are commonly referred to as ampullary adenocarcinomas. In this study, we compared the clinicopathological features of intra-ampullary and periampullary carcinomas, including survival outcomes.

Methods

This retrospective cross-sectional study was conducted at the Department of Pathology, Liaquat National Hospital. All radiologically suspected cases or biopsy-proven (endoscopic biopsy) cases of intra-ampullary/periampullary carcinoma were included in the study. All patients underwent surgical resection (Whipple’s procedure/pancreatoduodenectomy). The classification of intra-ampullary and periampullary carcinomas was performed according to the College of American Pathologists (CAP) guidelines.

Results

Among the 188 case studies, most (61.7%, n = 116) were males, with a median age of 55 years. Most tumors were of the pancreatobiliary subtype (57.4%, n = 108). Similarly, intra-ampullary carcinoma was more common than periampullary carcinoma (61.7% vs. 38.3%). Intra-ampullary carcinoma showed a higher extent of involvement of adjacent structures, a higher frequency of perineural invasion, and a higher nodal stage than periampullary carcinoma. Similarly, the median disease-specific survival of intra-ampullary carcinoma was significantly lower (46 months) than that of periampullary carcinoma (53.5 months).

Conclusion

We found a higher incidence of intra-ampullary carcinoma in our study. In addition, intra-ampullary carcinoma had a worse survival rate and was associated with poorer pathological parameters, such as perineural invasion and higher nodal and tumor stages than periampullary carcinoma.

## Introduction

The ampulla of Vater refers to the structure in the duodenal wall in which the biliary and pancreatic ducts open. It is lined by pancreatobiliary-type mucosa, and outside surfaces are lined by enteric-type mucosa. Malignant epithelial tumors arising at this site are commonly referred to as ampullary adenocarcinomas. These tumors are a heterogeneous group because of the complex nature of the ampulla [1]. Owing to clinical and histopathological differences, they are divided into intra-ampullary and periampullary carcinomas. Intra-ampullary carcinomas arise within the ampulla and are more likely to cause obstructive jaundice. Periampullary carcinoma arises from the duodenal surface of the papillae, and it is detected late. Because of the complex anatomy of the ampulla, it is often difficult to characterize these tumors. Moreover, it is important to differentiate these tumors from pancreatic ductal adenocarcinomas and bile duct cancers. Therefore, a careful gross examination is mandatory for definite categorization.

Various pathological parameters determine the prognosis of ampullary cancer. These include nodal metastasis, the extent of tumor involvement of adjacent structures, perineural invasion (PNI), and lymphovascular invasion (LVI) [2,3]. Moreover, there are two common histological types of ampullary carcinoma, pancreatobiliary and intestinal, with notable prognostic differences [4]. Studies have also shown differences in survival between intra-ampullary and periampullary carcinomas [1]. However, reports from different parts of the world regarding differences in survival among these tumor types are contradictory, and various parameters other than intrinsic tumor features, such as obstructive jaundice and stage of presentation, can affect survival. Therefore, devising treatment/management guidelines based on locoregional data are imperative. Therefore, in this study, we compared the clinicopathological features of intra-ampullary and periampullary carcinomas, including survival outcomes.

## Materials and methods

Study design, setting, and inclusion criteria

This retrospective cross-sectional study was conducted at the Department of Pathology, Liaquat National Hospital. All radiologically suspected or biopsy-proven (endoscopic biopsy) cases of ampullary/periampullary adenocarcinoma were included in the study. All patients underwent surgical resection (Whipple’s procedure/pancreatoduodenectomy). Surgical resections were performed at the Liaquat National Hospital from January 2019 to December 2023. Patients with incomplete surgical records, lack of clinical follow-up, or unavailability of histopathological data were excluded from the study. In addition, patients with tumors located in the head of the pancreas, bile ducts, or gallbladder, in addition to those receiving prior neoadjuvant chemotherapy or radiation, were excluded. The categorization of intra-ampullary and periampullary carcinomas was performed according to the College of American Pathologists (CAP) guidelines. Intra-ampullary carcinomas included those arising from the ampullary duct. In contrast, periampullary carcinomas included ampullary duodenal tumors arising from the duodenal surface of the papillae. Mixed ampullary and periampullary carcinomas were excluded from the study. Histological categorization into pancreatobiliary and intestinal subtypes was performed using routine hematoxylin and eosin staining with adjunctive caudal-type homeobox transcription factor 2 (CDX2) staining in all cases and cytokeratin 7 (CK7), cytokeratin 20 (CK20), and mucin (MUC) staining in a limited number of cases. Histological evaluation was performed by two senior histopathologists.

Pathological data and clinical follow-up

After surgical resection, pancreatoduodenectomy specimens were sent to the histopathology laboratory. A gross pathological examination was performed after formalin fixation. The dimensions of the stomach, duodenum, and pancreas were also noted. Surgical margins were inked, and representative pancreatic neck, retroperitoneal/uncinate (corresponding to superior mesenteric artery surface), common bile duct/common hepatic duct, and proximal (stomach) and distal (duodenal) margins were submitted. Moreover, the surfaces, including the vascular groove (corresponding to the superior mesenteric vein) and anterior pancreatic surface, were also inked with different colors. After securing the margins, the duodenum and stomach were opened along the greater curvature, and the ampullary area was thoroughly examined for any irregularity, mass, nodule, or color change. The pancreas was then opened along the common bile duct and pancreatic duct simultaneously up to the ampulla, and the exact location of the tumor (intra-ampullary vs. periampullary) was established. The tumor is submitted in entirety in relation to adjacent structures, along with representative sections of the stomach, duodenum, pancreas, bile duct, gall bladder (if present), and peri-pancreatic and peri-duodenal fat for the lymph nodes. Tumor size, location, and extent of involvement were noted. Histopathological findings were examined by experienced pathologists, and the final tumor classification, grade, and staging were performed. Clinical data on disease recurrence and survival were obtained from clinical records.

Statistical analysis

The data were analyzed using the IBM SPSS Statistics for Windows, Version 26 (Released 2019; IBM Corp., Armonk, New York, United States). The mean and standard deviation, median, and interquartile range of quantitative variables were calculated. Frequencies and percentages for qualitative variables were calculated. Chi-square and Fisher's exact tests were applied to determine the association between clinicopathological parameters and tumor subtypes. Kaplan-Meir survival curves were used for survival analysis.

## Results

Clinicopathological features of ampullary carcinomas

Of the 188 case studies, most (61.7%, n = 116) were male, with a median age of 55 years. Most tumors were well differentiated (53.2%, n = 100), with peripancreatic tissue involvement observed in 40.4% (n = 76) of cases. LVI, PNI, and nodal metastasis were observed in 27.7% (n = 52), 42.6% (n = 80), and 42.6% (n = 80) of cases, respectively. Most tumors were of the pancreatobiliary subtype (57.4%, n = 108). Similarly, intra-ampullary carcinoma was more common than periampullary carcinoma (61.7% vs. 38.3%), as shown in Table 1.

**Table 1:** Descriptive statistics of study population IQR:  Inter-quartile range; N: nodal. Data has been presented as median (IQR) and n (%)

Clinicopathological parameters	Values
Gender	
Female, n (%)	72 (38.3)
Male, n (%)	116 (61.7)
Age (years)	
Median (IQR)	55 (44-60)
Age groups	
≤50 years, n (%)	60 (31.9)
>50 years, n (%)	128 (68.1)
Tumor size (cm)	
Median (IQR)	2.70 (2.00-3.80)
Tumor size groups	
<2 cm, n (%)	32 (17)
2-5 cm, n (%)	140 (74.5)
>5 cm, n (%)	16 (8.5)
Follow up duration (months)	
Median (IQR)	44.00 (38.00-55.00)
Tumor grade	
Grade 1/well-differentiated, n (%)	100 (53.2)
Grade 2/moderately differentiated, n (%)	72 (38.3)
Grade 3/poorly differentiated, n (%)	16 (8.5)
Tumor extent	
Limited ampulla, n (%)	8 (4.3)
Invades muscularis propria, n (%)	72 (38.3)
Invades pancreas up to 0.5 cm, n (%)	4 (2.1)
Invades into peripancreatic soft tissues, n (%)	76 (40.4)
Invades into peri duodenal tissues, n (%)	28 (14.9)
Lymphovascular invasion	
Present, n (%)	52 (27.7)
Absent, n (%)	136 (72.3)
Perineural invasion	
Present, n (%)	80 (42.6)
Absent, n (%)	108 (57.4)
Nodal metastasis	
Present, n (%)	80 (42.6)
Absent, n (%)	108 (57.4)
Nodal (N) stage	
N0, n (%)	80 (42.6)
N1, n (%)	84 (44.7)
N2, n (%)	24 (12.8)
Tumor type	
Intestinal, n (%)	72 (38.3)
Pancreatobilliary, n (%)	108 (57.4)
Neuroendocrine differentiation, n (%)	8 (4.3)
Tumor site	
Intra-ampullary, n (%)	116 (61.7)
Periampullary, n (%)	72 (38.3)

Comparison of clinicopathological features of intra-ampullary and periampullary carcinoma

Table 2 compares the clinicopathological features of the two groups of ampullary carcinomas (intra-ampullary and periampullary). A significant association was noted with respect to tumor extent, PNI, and nodal metastasis. Intra-ampullary carcinoma showed a higher extent of involvement of adjacent structures, higher frequency of PNI, and higher nodal stage than periampullary carcinoma.

**Table 2:** Association of intra-ampullary and periampullary carcinoma with clinicopathological parameters N: nodal. Data has been presented as n (%). Chi-square/Fisher's exact test was applied. *p-value significant as <0.05

Clinicopathological parameters	Values		p-value
	Intra-ampullary	Periampullary	
Gender			
Female, n (%)	48 (41.4)	24 (33.3)	0.270
Male, n (%)	68 (58.6)	48 (66.7)	
Age groups			
≤50 years, n (%)	36 (31)	24 (33.3)	0.742
>50 years, n (%)	80 (69)	48 (66.7)	
Tumor size			
<2 cm, n (%)	24 (20.7)	8 (11.1)	0.174
2-5 cm, n (%)	84(72.4)	56 (77.8)	
>5 cm, n (%)	8 (6.9)	8 (11.1)	
Tumor grade			
Grade 1/well-differentiated, n (%)	60 (51.7)	40 (55.6)	0.514
Grade 2/moderately differentiated, n (%)	44 (37.9)	28 (38.9)	
Grade 3/poorly differentiated, n (%)	12 (10.3)	4 (5.6)	
Tumor extent			
Limited ampulla, n (%)	4 (3.4)	4 (5.6)	0.045*
Invades muscularis propria, n (%)	36 (31)	36 (50)	
Invades pancreas up to 0.5 cm, n (%)	4 (3.4)	0 (0)	
Invades into peripancreatic soft tissues, n (%)	52 (44.8)	24 (33.3)	
Invades into periduodenal tissues, n (%)	20 (17.2)	8 (11.1)	
Lymphovascular invasion			
Present, n (%)	28 (24.1)	24 (33.3)	0.171
Absent, n (%)	88 (75.9)	48 (66.7)	
Perineural invasion			
Present, n (%)	56 (48.3)	24 (33.3)	0.044*
Absent, n (%)	60 (51.7)	48 (66.7)	
Nodal metastasis			
Present, n (%)	68 (58.6)	40 (55.6)	0.679
Absent, n (%)	48 (41.4)	32 (44.4)	
Nodal (N) stage			
N0, n (%)	48 (41.4)	32 (44.4)	0.003*
N1, n (%)	60 (51.7)	24 (33.3)	
N2, n (%)	8 (6.9)	16 (22.2)	
Tumor type			
Intestinal, n (%)	48 (41.4)	24 (33.3)	0.464
Pancreatobilliary, n (%)	64 (55.2)	44 (61.1)	
Neuroendocrine differentiation, n (%)	4 (3.4)	4 (5.6)	

Survival (disease-specific survival) analysis of intra-ampullary and periampullary carcinoma

Table 3 shows that the median disease-specific survival of intra-ampullary carcinoma was significantly lower (46 months) than that of periampullary carcinoma (53.5 months).

**Table 3:** Means and medians for disease-specific survival (Intra-ampullary and periampullary carcinoma) CI: confidence interval. Data has been presented as mean (standard deviation) and median (inter-quartile range). p-value significant as <0.05

Tumor type	Number of events	Mean (95% CI)	Median (95% CI)	Log rank p-value
Intra-ampullary (n = 116)	72	46.08 (44.075-48.085)	46.000 (43.369-48.631)	<0.001*
Periampullary (n = 72)	32	53.538 (51.323-55.754)	57.000 (54.789-59.211)	
Overall (n = 188)	104	48.818 (47.236-50.40)	53.000 (48.89-57.11)	

**Figure 1:**
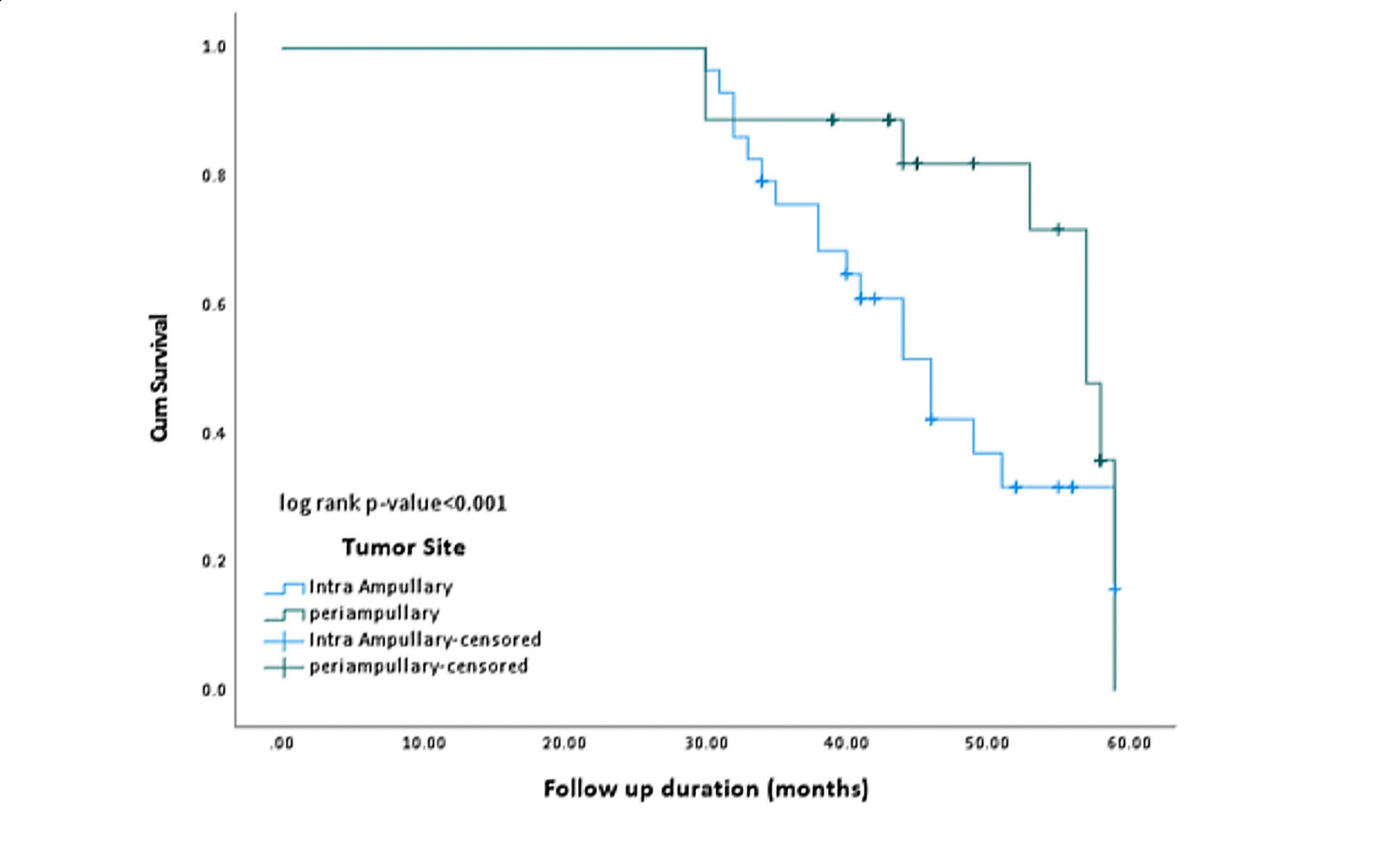
Kaplan-Meier curve for disease-specific survival (intra-ampullary and periampullary carcinoma)

Figure 1 depicts the comparison of survival analysis of these two groups of tumors (intra-ampullary and periampullary) using Kaplan-Meier curves. Disease-specific survival was better in patients with periampullary carcinoma than in those with intra-ampullary cancer (p < 0.001). 

## Discussion

In this study, we evaluated the clinicopathological features and survival analysis of intra-ampullary and peri-ampullary carcinomas and found that intra-ampullary carcinomas were significantly associated with poor pathological parameters, such as PNI and higher disease stage. Similarly, intra-ampullary carcinoma showed lower disease-specific survival than peri-ampullary carcinoma.

Adsay et al. [1] recommended four distinct subtypes of ampullary adenocarcinoma: invasive carcinoma arising from intra-ampullary papillary-tubular neoplasm (IAPN), ampullary ductal adenocarcinoma, and peri-ampullary duodenal and ampullary carcinoma not otherwise specified. Among these tumors, the first two categories were collectively labeled intra-ampullary carcinoma, as per the CAP protocol. Adsay et al. reported different pathological features and outcomes of ampullary carcinoma arising from IAPN and ampullary ductal. They reported the worst outcomes of ampullary ductal carcinoma, with a three-year survival rate of 41%. Because most intra-ampullary carcinomas are ampullary ductal, these findings correlate with our study findings, emphasizing the worst outcome of intra-ampullary carcinomas. Contrary to these findings, a few authors have questioned the distinction between ampullary carcinomas and other pancreatobiliary carcinomas of the same histology. Westgaard et al. [5] conducted a study involving 207 pancreatoduodenectomy specimens and revealed that ampullary carcinomas have the same long-term outcomes as pancreatic, duodenal, and biliary carcinomas of the same histological type. Therefore, the authors emphasized the importance of pancreatobiliary and intestinal differentiation. They found that 63% of their patients had pancreatobiliary differentiation. We also found the pancreatobiliary subtype to be the most common histological type (57.4%).

There are two main histological subtypes of ampullary carcinomas: pancreatobiliary and intestinal. Previous studies have reported poorer outcomes in patients with pancreatobiliary than intestinal carcinoma [6,7]. Our results showed that 55.2% of intra-ampullary carcinomas had a pancreatobiliary histological subtype, indicating the importance of histological typing in ampullary carcinoma.

Multiple studies have emphasized that ampullary carcinomas have better outcomes than pancreatic ductal adenocarcinomas [8-10]. The primary reason was a relatively early presentation of ampullary carcinoma associated with biliary obstruction. Conversely, pancreatic cancers grow insidiously and present late in the disease course. We did not compare outcomes between pancreatic cancer and ampullary carcinoma.

Despite different clinical outcomes and distinct nomenclature, it remains controversial whether intra-ampullary and peri-ampullary carcinomas are biologically and genetically distinct. Tumor protein 53 (TP53), Kirsten rat sarcoma virus protein (KRAS), breast cancer type 1 and 2 susceptibility proteins (BRCA1/2), mothers against decapentaplegic homolog 4 (SMAD4), and microsatellite instability-related molecular alterations are described in ampullary carcinomas, and these also seem to correlate with histological subtypes, i.e., pancreatobiliary and intestinal [11]. In our study, we did not evaluate the molecular features of ampullary carcinoma.

Limitations

A major limitation of our study was the lack of molecular evaluation of intra-ampullary and peri-ampullary carcinomas. It is important to compare the molecular features of these two groups of tumors to understand whether the difference in survival is based only on tumor location or genetic characteristics. Second, given that this was a single-center study conducted at a private hospital, the results cannot be extrapolated to the entire population; therefore, multicenter studies are needed, especially those involving public sector hospitals, to specifically understand the survival difference between these two groups of tumors.

## Conclusions

In this study, we found that intra-ampullary carcinomas comprise most ampullary carcinomas. In addition, intra-ampullary carcinomas showed poorer disease-specific survival than peri-ampullary carcinomas. Moreover, intra-ampullary carcinomas are associated with worse prognostic parameters, such as extent of involvement, nodal metastasis, and PNI.
